# Evaluating Silent Reading Performance with an Eye Tracking System in Patients with Glaucoma

**DOI:** 10.1371/journal.pone.0170230

**Published:** 2017-01-17

**Authors:** Noriaki Murata, Daiki Miyamoto, Tetsuya Togano, Takeo Fukuchi

**Affiliations:** 1 Division of Ophthalmology and Visual Science, Graduated School of Medical and Dental Sciences, Niigata University, Niigata, Japan; 2 Department of Orthoptics and Visual Sciences, Faculty of Medical Technology, Niigata University of Health and Welfare, Niigata, Japan; Bascom Palmer Eye Institute, UNITED STATES

## Abstract

**Objective:**

To investigate the relationship between silent reading performance and visual field defects in patients with glaucoma using an eye tracking system.

**Methods:**

Fifty glaucoma patients (Group G; mean age, 52.2 years, standard deviation: 11.4 years) and 20 normal controls (Group N; mean age, 46.9 years; standard deviation: 17.2 years) were included in the study. All participants in Group G had early to advanced glaucomatous visual field defects but better than 20/20 visual acuity in both eyes. Participants silently read Japanese articles written horizontally while the eye tracking system monitored and calculated reading duration per 100 characters, number of fixations per 100 characters, and mean fixation duration, which were compared with mean deviation and visual field index values from Humphrey visual field testing (24–2 and 10–2 Swedish interactive threshold algorithm standard) of the right versus left eye and the better versus worse eye.

**Results:**

There was a statistically significant difference between Groups G and N in mean fixation duration (G, 233.4 msec; N, 215.7 msec; *P* = 0.010). Within Group G, significant correlations were observed between reading duration and 24–2 right mean deviation (*r*_*s*_ = -0.280, *P* = 0.049), 24–2 right visual field index (*r*_*s*_ = -0.306, *P* = 0.030), 24–2 worse visual field index (*r*_*s*_ = -0.304, *P* = 0.032), and 10–2 worse mean deviation (*r*_*s*_ = -0.326, *P* = 0.025). Significant correlations were observed between mean fixation duration and 10–2 left mean deviation (*r*_*s*_ = -0.294, *P* = 0.045) and 10–2 worse mean deviation (*r*_*s*_ = -0.306, *P* = 0.037), respectively.

**Conclusions:**

The severity of visual field defects may influence some aspects of reading performance. At least concerning silent reading, the visual field of the worse eye is an essential element of smoothness of reading.

## Introduction

Glaucoma is a leading cause of acquired blindness worldwide [1, 2]. Glaucoma often reduces quality of life (QOL) by making daily activities difficult, such as walking, driving, and reading [3–5]. There are two main methods to quantify reading in ophthalmic patients: questionnaires [6–12] and reading function tests. Questionnaires can assess a broad tendency towards reading difficulty in daily life. Many reading tests have been developed [13], such as the Bailey-Lovie Near Reading Card [14] the Pepper Visual Skills for Reading Test (VSRT) [15], Minnesota Low-Vision Reading Test (MNREAD) [16], the International Reading Test (IReST) [17], and the Rapid Serial Visual Presentation (RSVP) [18]. These tests have been used in studies of reading assessment in patients with glaucoma [19–21]. Clinical reading tests are thoroughly standardized and highly artificial [13], so they are superior in precisely quantifying the reading ability of patients. However, reading tests have limitations in measuring reading ability because they are conducted in artificial settings. Furthermore, clinical reading tests use spoken reading to evaluate reading quality, but evaluation of spoken reading assesses a combination of speaking and reading performance. It is possible that tests of spoken reading do not reflect actual reading situations because we have few opportunities to read sentences or characters in newspapers, magazines, novels, and product labels aloud in our daily lives. Reading involves tasks not measured in standard reading tests [22], and clinical reading tests are not able to evaluate the performance of “browse” reading that quickly gathers information from documents. Consequently, it is necessary to focus on evaluating silent reading ability. Recently, several studies have reported silent reading performance in patients with glaucoma using an eye tracking system [23–25]. An eye tracking system is capable of quickly and non-invasively measuring human gaze and eye movements. Previously, relationships between glaucomatous visual field (VF) defects and facial recognition [26] and driving in patients with glaucoma have been studied using an eye tracking system [27, 28]. The biggest advantage of using an eye tracking system to evaluate reading performance is the ability to measure eye movements when participants read silently. In addition, it is capable of choosing the stimulus sentences freely. Smith et al. [23] compared reading performance in the better and worse eyes, defined by laterality of VF defects in patients with glaucoma. Burton et al. [24] have examined differences in reading performance with both eyes between healthy subjects and patients with advanced glaucoma. They have also investigated the relationship between reading speed [25] and integrated visual field (IVF) in patients by taking the best sensitivity values from corresponding VF locations from both eyes [29, 30]. Some researchers have described different research methods for assessing silent reading in patients with glaucoma, yet the most appropriate measure of silent reading has not yet been determined. The influence of glaucomatous VF defects on silent reading and what VF scores contribute to reading difficulty remain unclear. The aim of the current study is to compare differences in reading performance among healthy participants and patients with glaucoma. Furthermore, using an eye tracking system, we investigate the relationship between silent reading and glaucomatous VF defects as indicated by indices routinely used in daily medical practice.

## Methods

### Participants

This prospective cross-sectional study was approved by the ethics committee of the Niigata University Graduate School of Medical and Dental Science. We followed the tenets of the Declaration of Helsinki. The individual in this manuscript has given written informed consent (as outlined in PLOS consent form) to publish these case details. All data were transferred to a secure computer at the university with patient identifiers removed.

Patients with glaucoma were recruited from Niigata University Medical and Dental Hospital, in Niigata, Japan. All patients in the database had undergone a routine comprehensive ophthalmic examination that included assessment of best-corrected visual acuity (BCVA) using a 5-m Landolt chart, refraction, keratometry, slit-lamp examination, Goldmann applanation tonometry, gonioscopy, indirect ophthalmoscopy, dilated slit-lamp optic disc examination, VF testing using the 24–2 Swedish Interactive Threshold Algorithm (SITA) Standard Strategy (Humphrey Field Analyzer: HFA; Carl Zeiss Meditec, Inc., Dublin, CA, USA) and the 10–2 SITA Standard Strategy, and spectral domain optical coherence tomography (OCT) examination with the 3D-OCT 2000 (Topcon, Inc., Tokyo, Japan). Clinical diagnosis of glaucoma was based on European Glaucoma Society [31] and Japan Glaucoma Society [32] guidelines. All recruited patients had early to advanced VF defects in either the right eye, left eye, or both eyes, but BCVA was better than ±0.00 logarithm of the minimum angle of resolution (logMAR), which is equivalent to Snellen 20/20 in both eyes. The definition and classification of glaucomatous VF defects were based on the Anderson and Patella criteria [33]. Ophthalmic data collected on patients with glaucoma included BCVA, 24–2 and 10–2 mean deviation (MD), 24–2 visual field index (VFI), and 10–2 MD. VF results that were reliable, defined as fixation loss <20%, false-positive rate <15%, and false-negative rate <33%, were included in the analysis. VF scores were categorized as from the right eye or left eye (24–2 right MD, 24–2 left MD, right VFI, left VFI, 10–2 right VFI, and 10–2 left VFI). The better and worse eyes were compared by MD and VFI (24–2 better MD, 24–2 worse MD, better VFI, worse VFI, 10–2 better MD, and 10–2 worse MD).

Healthy control participants composed of medical staff with BCVA of at least ±0.00 logMAR were included in the current study. We performed 24–2 SITA fast for all controls to confirm that they had no VF defects. A participant with a Glaucoma Hemifield Test Classification of ‘within normal limits’ was included as a control.

For all participants, Japanese was the first language. Participants were between 19 and 70 years of age. We recruited participants with educational attainment greater than or equal to Japanese high-school graduates. Participants were not recruited if they had a diagnosed reading difficulty such as dyslexia. Participants with refractive error greater than or equal to ±6.0 diopters (spherical equivalent), astigmatic error greater than or equal to ±2.5 diopters, or amblyopia in either eye were excluded. All potential participants had slit-lamp biomicroscopy performed by an ophthalmologist before the reading experiment. They were excluded if they had ocular surface disease or complications other than glaucoma.

### Reading Experiment

**Fig 1:**
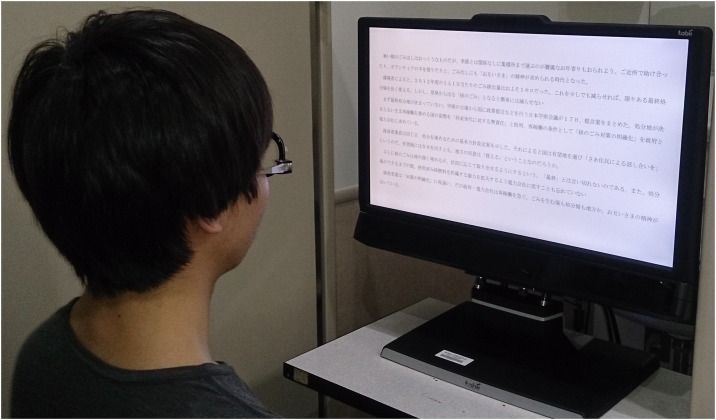
Tobii Tx300 setup during the reading experiment. Participants were evaluated during silent reading without a forehead and a chin rest in order to reproduce reading conditions in daily life. Participants were instructed not to lean forward in order to maintain the test distance. It is important to note that our reading experiment included the process of measuring either the point of gaze or the motion of an eye relative to the head. Therefore, we excluded data during saccades from the analysis of reading performance.

Eye movements were monitored using a Tobii TX300 eye tracking device (Tobii Technology, Danderyd, Sweden) with an accuracy of 0.4° and a sampling rate of 300 Hz. The stimuli were three articles composed of 607–612 characters with 12–13 lines per paragraph from a column named *Nipposho* from the *Niigata Nippo* newspaper, used with permission. All texts were fixed and non-scrolling and written horizontally. Readability was determined to be at a ninth-grade level using the *Obi2* tool [34]. TX300 is an integrated eye tracker with a removable 23-inch thin-film transistor liquid crystal display monitor. The stimuli were presented in a dedicated display at a resolution of 1920×1080 pixels, with the refresh rate of 60 Hz. They were also displayed in black letters on a white background, and the luminance of the white background was fixed at 140 cd/m^2^. The illuminance of the experimental room was adjusted between 400 and 600 lux. The distance from eye tracker to the participants was checked at the beginning of the experiment when calibration was performed for the eye tracker. The test distance between the participant and the monitor was 60 cm. The text was displayed in 22-point Mincho font, in which each letter subtended a maximum height of 0.735° visual angle. Participants asked to read “as if you are reading a novel or newspaper in your daily life, as quickly and accurately as possible” when they performed silent reading using both eyes with appropriate refractive correction (Fig 1).

### Eye Tracking Data Processing

**Fig 2:**
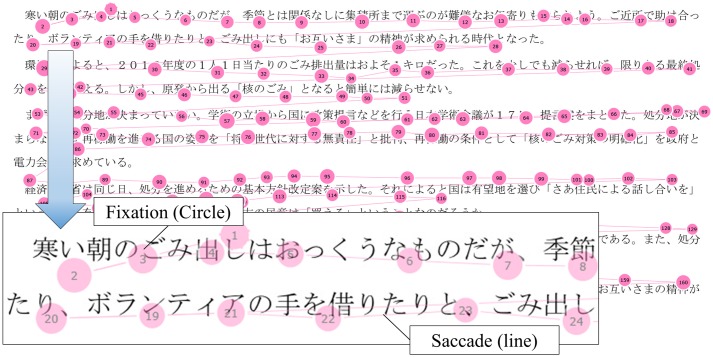
A participant’s gaze pattern during the reading experiment. The articles contain both Japanese syllabary (*Hiragana*) and Chinese characters (*Kanji*). A participant reads sentences in a horizontal direction from left to right. The size of the circle corresponds to the fixation duration. The number in the circle is the rank of fixation. The lines represent saccades.

Data from the eye tracker was extracted using the Tobii Studio appurtenance software, which obtained data on total visit duration, fixation count, and total fixation duration within a designated area of articles. An example of fixation and saccades over an article is provided in Fig 2. For each participant, we adopted three parameters to evaluate reading performance. Reading duration per 100 characters (RD) was calculated by dividing total fixation duration by character count ×100. Number of fixation time per 100 characters (NF) was calculated by dividing fixation count by character count ×100. Mean fixation duration (mFD) was calculated by dividing total fixation duration by fixation count. These values were analyzed for each sentence, and reading parameters for a participant were calculated by averaging values from the three articles.

### Statistical Analysis

Statistical analysis was performed using SPSS version 21.0 (IBM Corp., Armonk, NY, USA). To assess differences in age, gender, and reading performance between patients with glaucoma and control participants, we used the Mann-Whitney U-test, chi-square test, and Student’s t-test as appropriate. Spearman’s rank correlation coefficients were used to evaluate associations between reading parameters and glaucomatous VF defects measured by the HFA 24–2 and 10–2 programs. *P* values < 0.05 were considered statistically significant. We defined correlations as “good” when the correlation coefficient was between 0.4 and 0.6, “moderate” when between 0.2 and 0.39, and “poor” when less than 0.2.

## Results

### Comparison of Patients with Glaucoma and Controls

Fifty patients with glaucoma (Group G) and 20 healthy normal controls (Group N) were included in this study. There was a significant difference in mFD between patients and controls (G, 233.4 msec; N, 215.7 msec; *P* = 0.010). However, no significant differences were noted in RD (G, 9.4 sec; N, 8.9 sec; *P* = 0.543) and NF (G, 33.0 times; N, 32.7 times; *P* = 0.925). The demographic characteristics of the study participants and the comparison of reading parameters between the two groups are summarized in Table 1.

**Table 1:** Demographic characteristics and reading parameters in healthy controls and patients with glaucoma.

Variables	Healthy controls (*n* = 20)	Patients with glaucoma (*n* = 50)	*P* value for between-group difference
Age, mean ± SD	46.9 ± 17.2	52.2 ± 11.4	0.482*
Sex, male:female	10:10	30:20	0.445†
RV, logMAR	-0.071 ± 0.024	-0.079 ± 0.000	0.198‡
LV, logMAR	-0.071 ± 0.024	-0.079 ± 0.000	0.146‡
RD (sec)	8.8 ± 3.4	9.4 ± 3.3	0.543‡
NF (times)	32.7± 10.6	33.0 ± 10.7	0.925‡
mFD (msec)	215.7 ± 24.9	233.4 ± 25.1	0.010‡

Abbreviations: logMAR, logarithm of the minimum angle of resolution; LV, left visual acuity; mFD, mean fixation duration; NF, number of fixation per 100 characters; RD, reading duration per 100 characters; RV, right visual acuity; SD, standard deviation

* Mann-Whitney U-test

^†^ Chi-square test

^‡^ Student’s t-test.

### Correlations between Reading Performance and VF

VF defect scores in patients with glaucoma are summarized in Table 2. Three patients were removed from the 10–2 analysis because of exclusion criteria or unacceptable reliability. Significant correlations were observed between RD and 24–2 right MD (*r*_*s*_ = -0.280, *P* = 0.049), 24–2 right VFI (*r*_*s*_ = -0.306, *P* = 0.030), 24–2 worse VFI (*r*_*s*_ = -0.304, *P* = 0.032), and 10–2 worse MD (*r*_*s*_ = -0.326, *P* = 0.025). Significant correlations were observed between mFD and 10–2 left MD (*r*_*s*_ = -0.294, *P* = 0.045) and 10–2 worse MD (*r*_*s*_ = -0.306, *P* = 0.037), respectively. No significant correlation was found between FT and any VF scores. There were no significant correlations observed between 24–2 left MD, 24–2 better MD, 24–2 worse MD, 24–2 left VFI, 24–2 better VFI, 10–2 Right MD, 10–2 better MD, and any reading parameters. Results of statistical comparisons in patients with glaucoma are summarized in Table 3.

**Table 2:** MD and VFI scores of patients with glaucoma.

Eye	Index	Test	Mean ± SD	Range
Right	MD	24–2 (dB)	-13.07 ± 8.46	-1.36 – -29.00
Left	MD	24–2 (dB)	-13.03 ± 7.69	-1.29 – -28.35
Better	MD	24–2 (dB)	-9.53 ± 7.34	-1.29 – -25.73
Worse	MD	24–2 (dB)	-16.57 ± 7.18	-4.89 – -29.00
Right	VFI	24–2 (%)	62.76 ± 26.11	99.00 – 12.00
Left	VFI	24–2 (%)	64.56 ± 23.77	100.00 – 10.00
Better	VFI	24–2 (%)	73.68 ± 22.51	100.00 – 25.00
Worse	VFI	24–2 (%)	53.64 ± 23.17	93.00 – 10.00
Right	MD	10–2 (dB)	-12.30 ± 8.78	-0.67 – -30.39
Left	MD	10–2 (dB)	-11.06 ± 7.09	-0.18 – -26.06
Better	MD	10–2 (dB)	-8.51 ± 7.12	-0.18 – -25.85
Worse	MD	10–2 (dB)	-14.86 ± 7.54	-1.60 – -30.39

Abbreviations: 10–2, 10–2 SITA standard field test; 24–2, 24–2 SITA standard field test; MD, mean deviation; VFI, visual field index.

**Table 3:** Spearman’s rank correlation coefficients comparing reading parameters and visual field defects.

**24–2**	**Better MD**		**Worse MD**		**Right MD**		**Left MD**	
	*P*	(*r*_*s*_)	*P*	(*r*_*s*_)	*P*	(*r*_*s*_)	*P*	(*r*_*s*_)
RD	0.743	(-0.047)	0.054	(-0.273)	0.049*	(-0.280)	0.958	(-0.007)
NF	0.635	(0.069)	0.204	(-0.182)	0.195	(-0.186)	0.525	(0.092)
mFD	0.241	(-0.169)	0.050	(-0.279)	0.150	(-0.206)	0.140	(-0.212)
**24–2**	**Better VFI**		**Worse VFI**		**Right VFI**		**Left VFI**	
RD	0.677	(-0.060)	0.032*	(-0.304)	0.030*	(-0.306)	0.768	(-0.043)
NF	0.709	(0.054)	0.114	(-0.226)	0.135	(-0.214)	0.734	(0.049)
mFD	0.215	(-0.178)	0.056	(-0.272)	0.084	(-0.247)	0.180	(-0.193)
**10–2**	**Better MD**		**Worse MD**		**Right MD**		**Left MD**	
	*P*	(*r*_*s*_)	*P*	(*r*_*s*_)	*P*	(*r*_*s*_)	*P*	(*r*_*s*_)
RD	0.432	(-0.117)	0.025*	(-0.326)	0.032	(-0.215)	0.147	(-0.220)
NF	0.946	(0.010)	0.131	(-0.224)	0.503	(-0.100)	0.232	(-0.182)
mFD	0.072	(-0.265)	0.037*	(-0.306)	0.104	(-0.240)	0.045*	(-0.294)

Statistically significant associations are marked with asterisks.

Abbreviations: 10–2, 10–2 SITA standard field test; 24–2, 24–2 SITA standard field test; MD, mean deviation; mFD, mean fixation duration; NF, number of fixation per 100 characters; RD, reading duration per 100 characters; VFI, visual field index.

## Discussion

In this study, we evaluated reading performance under conditions resembling daily activity of patients with glaucoma and controls using the Tobii TX300 eye tracking system. Patients with glaucoma had statistically significantly longer mFD. Thus, the presence of glaucomatous VF defects was considered to contribute to increased mFD. We assumed that the reading performance of patients with glaucoma was affected by VF defects even if there is no decline in visual acuity because we restricted study participants to glaucoma patients with VF defects but good BCVA. On the other hand, there was no significant difference in FT between Groups G and N. We inferred that patients and controls had similar spans of recognition. A previous experiment provided the minimum VF size necessary for rapid normal reading, which is called the critical VF size [35]. Critical VF size decreases when VF size ranges from 8° to 2°. Saccades became relatively small and the traced distance along one line stretched towards both ends. Consequently, reading time increases. According to a previous experiment, a similar span of recognition in patients and controls might result from including only participants with good visual acuity, or including patients with early to moderate glaucoma. Furthermore, we examined the relationship between reading parameters and MD or VFI values in the right or left eye and the better or worse eye. There were significant correlations between RD and 24–2 right MD, 24–2 right VFI, 24–2 worse VFI, and 10–2 worse MD. Moreover, significant correlations were observed between mFD and 10–2 left MD and 10–2 worse MD, respectively. Therefore, even without calculating special VF parameters such as IVF-MD, it may be possible to predict reading difficulties in patients with glaucoma from data gathered in routine clinical examinations. However, only moderate correlations were observed between such indices of VF defects and reading performance.

Burton et al. [24] have previously reported that some patients with advanced VF defects read slower than controls. They used short text passages and eye tracking, but no significant differences in sentence reading speed was observed between the two groups. Based on their results, they speculated that reading speed in glaucoma is affected by the latency of short text passages. Our study found that mFD was prolonged in patients, even though RD was not different from controls. Ikeda and Saida [35] indicated some information pre-processing occurs in the peripheral VF, which eventually minimizes the pause duration at every fixation. These findings suggest that once patients have glaucomatous VF defects, it may affect the ability to recognize words or characters.

Smith et al. [23] reported that mean reading duration and saccade rate were significantly different in the worse eye compared with the better eye during monocular reading. Eye movement indicating re-reading a previous section and not conforming to expected patterns were frequently observed in the worse eye. It was evident that glaucomatous VF defects lead to lower monocular reading performance. On the other hand, in the current study, we demonstrated a correlation between reading parameters with both eyes. Some parts of the VF defect scores measure monocular programs, hence it follows that silent reading performance with both eyes is affected by VF defects of either the left or right eye.

Comparisons between the better and worse eyes in our current study demonstrate that 10–2 worse MD, RD, and mFD are correlated, as well as worse VFI and RD. Statistical associations were found in the only score that emphasizes the central VF. Accordingly, we hypothesize that the severity of central VF defects contributes to a decline in reading performance. Whittaker et al. [36] reported that VF defects that are close to fixation inhibit reading to a greater extent than peripheral VF defects. It is possible that findings from the analysis of better and worse eyes in the current study reflect the previous theory. A previous study has demonstrated that out-loud reading performance in particular was affected by severe VF loss of the better eye in patients with glaucoma [22]. When Ishii et al. [20] evaluated out-loud reading performance of Japanese patients with glaucoma using MNREAD-J, they observed that patients had slower maximum reading speed, larger critical size point, and lower reading acuity than normal subjects. Moreover, their study demonstrated a relationship between MD in the 30–2 and 10–2 programs in the better eye and critical size point. Thus, they speculated that reading performance in patients might mostly rely on the visual function of the better eye. On the other hand, a study that examined the correlation between Japanese version of the 25-item National Eye Institute Visual Function Questionnaire (NEI VFQ-25) [37] questionnaire score and 30–2 program MD [38] has found correlations with MD in the better eye and the worse eye. In addition, they found a slightly stronger relationship between the better eye, QOL, and visual function. Therefore, they speculated that the better eye might contribute more to QOL. However, a recent study of silent reading using an eye tracker observed no significant association between IVF-MD and reading speed [25]. IVF-MD values calculated by taking the best sensitivity are probably dependent on the sensitivity of the better eye. In the current study, no significant correlations between MD and VFI score of the better eye or reading performance with both eyes were observed. Thus, these results are not inconsistent with previous research using IVF-MD values. Although previous questionnaire-based studies and studies that evaluated oral reading in patients with glaucoma have suggested that reading performance is directly affected by VF defects of the better eye, our findings indicate that VF of the worse eye might also be involved as an essential element, at least for silent reading. Binocular VF is not simply masked by the visual sensitivity of the better eye during silent reading. Reading in daily life is influenced not only by the better eye; the worse eye plays a role.

In the current study, we studied the correlation between right or left eye and reading performance. A statistically significant moderate correlation between right 24–2 MD, right VFI, and RD was observed, as well as between 10–2 left MD and mFD. Mishkin et al. [39] have demonstrated that subjects recognized significantly more English words placed in certain parts of the right VF than in the corresponding part of the left using a tachistoscope. Although we use horizontally written Japanese articles in the current study, reading direction is from left to right, the same as in English. It is possible that a part of reading performance, guessing the meaning from the context, was reduced due to VF defects in the right eye, as represented by 24–2 MD and VFI in the current study. Moreover, the right visual hemifield of the left eye tends to be affected by typical glaucomatous VF defects such as scotoma in Bjerrum’s area or the nasal step. Consequently, we speculate that the paracentral VF of the left eye contributes to increased mFD.

Our study has several limitations. First, it is conducted in Japanese. All previous reports of silent reading performance using eye trackers were conducted in English. Comparison of different languages in the same subject was difficult because it has been reported that Japanese students needed longer FD for reading English than Japanese texts [40]. The modern written Japanese consists of a combination of three character types: Chinese characters (*Kanji*), Japanese syllabary (*Hiragana*), and Latin script alphabet (*Romaji*). Therefore, it should be noted that reading evaluation using English articles and Japanese texts cannot simply be compared. Second, the Japanese writing system includes horizontal and vertical writing. In particular, Japanese newspapers are almost always printed vertically; it is important to also evaluate vertical writing in order to examine silent reading during daily activities in detail. Third, we evaluated the correlation between reading performance and VF defects values such as 24–2 MD, VFI, and 10–2 MD in the current study. The association between some reading parameters and visual field defects were indicated. However, details of an immediate cause of reading impairment are still unknown. In the future study, we are planning to perform multivariate analysis using variables such as visual acuity, visual field, age, gender, educational level, reading habits, viewing distance in daily life to find the cause of reading difficulties in glaucoma patients. Moreover, Fujita et al. [19] reported that patients with absolute scotoma within 3° of the central VF had difficulty reading when the scotoma involves more than two adjacent quadrants. Several studies using questionnaires analyzed not only the entire VF but also partial VFs measured by HFA. A study using the NEI VFQ-25 in Japanese patients with glaucoma [41] demonstrated the strongest correlation between NEI VFQ-25 scores and the lower paracentral VF in the better eye. A study using the Sumi questionnaire [10, 42] also noted that the inferior field is the most important area for near work such as reading and writing. Further experiments would be required to assess the associations between reading performance and more detailed VF quantification, for example, consideration of each point of the total deviation or using a clustered VF [43, 44] to evaluate which part of the VF is important in silent reading. It is necessary to evaluate combinations of subjective reading disability and objective eye movements during reading.

In conclusion, we observed that word or character recognition is prolonged in patients with glaucoma. Furthermore, monocular VF defects scores were correlated with lower reading performance in both eyes as evaluated using the Tobii TX300 eye tracking system. To understand the causes of reading impairment in the daily lives of patients with glaucoma, further study should promote assessment of silent reading under a particular set of conditions.
